# Understanding Lung Carcinogenesis from a Morphostatic Perspective: Prevention and Therapeutic Potential of Phytochemicals for Targeting Cancer Stem Cells

**DOI:** 10.3390/ijms22115697

**Published:** 2021-05-27

**Authors:** Win Sen Heng, Frank A. E. Kruyt, Shiau-Chuen Cheah

**Affiliations:** 1 Faculty of Medical Sciences, University Medical Center Groningen, 9713 GZ Groningen, The Netherlands; h.win.sen@umcg.nl (W.S.H.); f.a.e.kruyt@umcg.nl (F.A.E.K.); 2 Faculty of Medicine and Health Sciences, UCSI University, Kuala Lumpur 56000, Malaysia

**Keywords:** lung carcinogenesis, phytochemicals, cancer stem cell, epigallocatechin-3-gallate, sulforaphane

## Abstract

Lung cancer is still one of the deadliest cancers, with over two million incidences annually. Prevention is regarded as the most efficient way to reduce both the incidence and death figures. Nevertheless, treatment should still be improved, particularly in addressing therapeutic resistance due to cancer stem cells—the assumed drivers of tumor initiation and progression. Phytochemicals in plant-based diets are thought to contribute substantially to lung cancer prevention and may be efficacious for targeting lung cancer stem cells. In this review, we collect recent literature on lung homeostasis, carcinogenesis, and phytochemicals studied in lung cancers. We provide a comprehensive overview of how normal lung tissue operates and relate it with lung carcinogenesis to redefine better targets for lung cancer stem cells. Nine well-studied phytochemical compounds, namely curcumin, resveratrol, quercetin, epigallocatechin-3-gallate, luteolin, sulforaphane, berberine, genistein, and capsaicin, are discussed in terms of their chemopreventive and anticancer mechanisms in lung cancer and potential use in the clinic. How the use of phytochemicals can be improved by structural manipulations, targeted delivery, concentration adjustments, and combinatorial treatments is also highlighted. We propose that lung carcinomas should be treated differently based on their respective cellular origins. Targeting quiescence-inducing, inflammation-dampening, or reactive oxygen species-balancing pathways appears particularly interesting.

## 1. Introduction

Lung cancer is a malignant disease comprising two major histological categories known as non-small cell lung carcinoma (NSCLC) and small cell lung carcinoma (SCLC). In NSCLC, the major subtypes include adenocarcinoma (ADC), squamous cell carcinoma (SCC), and large cell carcinoma (LCC). A great proportion of cancer mortality today is still accounted for by lung cancer-related deaths (mainly NSCLC), representing 18.4% of all cancer deaths [1]. If proper preventive measures are not taken, about 1 in 5 people in countries with poor access to healthcare will die due to cancer by 2025 [2]. Some of the promoted cancer preventive measures include cessation of tobacco product and alcohol usage, pollution reduction, sufficient physical activity, and a healthy diet [3]. Among the proposed preventive solutions, eating a healthy diet that is mainly plant-based should be of primary concern, especially in developed countries, considering its huge contribution to overall health [4]. However, poor commitment due to various reasons such as high cost, poor taste, and low obtainability often renders this less achievable.

We generally acquire our nutrients from food in the form of macronutrients (i.e., carbohydrates, proteins, and fats) and micronutrients (i.e., vitamins and minerals). A plant-based diet that mainly consists of vegetables and fruits is considered good for well-being, mainly because it is enriched with vitamins, minerals, and dietary fibers. However, the contribution of micronutrients in vegetables and fruits to lowering the risk of lung cancer is controversial due to some inconsistent associations between the two variables [5,6]. Dietary fiber mainly helps in controlling other noncommunicable diseases such as cardiovascular disease, diabetes, and obesity [7]. Phytochemical compounds—the additional phytonutrients in vegetables and fruits—were found to have preventive and therapeutic potential for lung cancer as evidently shown by numerous in vitro and in vivo studies [8]. This suggests that phytochemical compounds may play a role in providing a plant-based diet’s added health benefits.

Cancer stem cell (CSC) theory postulates that there exists a tumor subpopulation for tumor maintenance, such as normal stem cells maintaining tissue steady state. CSC is the tumorigenic subpopulation capable of generating both tumorigenic and less tumorigenic offspring in the tumor bulk and is deemed responsible for the initiation and propagation of malignant diseases, including lung cancer [9]. This subpopulation is the reason why lung cancer gains resistance towards therapy over time and disease recurs in patients after initial remission [10]. Therefore, it is imperative to develop new therapies to target this subpopulation in order to achieve complete remission.

Hence, it is our goal to review the most recent findings on the benefits offered by plant-derived compounds regarding their potential to prevent or treat lung cancer. The mechanisms of action known thus far are discussed, such as modulation of inflammatory responses, oxidative stress, and CSCs. Firstly, we provide a comprehensive overview of lung functioning and relate this information with the pathological dysregulations of lung cancer at molecular and cellular levels. Subsequently, we focus on the relevant molecular pathways and relate them to potential phytochemical compounds, and we make comments based on the usefulness of targeting.

## 2. A Close Connection between Normal Lung Tissue Repair Mechanism and Carcinogenesis

The close resemblance of normal stem cells and CSCs is increasingly being recognized [11]. Both compartments are located at the apex of the hierarchy and possess high potency to proliferate and differentiate, thus making them capable of symmetric and asymmetric divisions. Both compartments also make use of respective stem cell niches for expansion cues and utilize similar signaling pathways in a tissue-specific manner [12,13]. Recently, we highlighted that lung progenitor cells could be the cells of origin of CSCs for different histological types of lung carcinomas, both of which are triggered to proliferate extensively in response to chronic injury [9]. In brief, basal cells, pulmonary neuroendocrine cells (PNECs), and alveolar epithelial cells II (AECs) are considered to be the cell origins of CSCs for SCC, SCLC, and ADC, respectively. It is not completely clear how these cells could evade a carcinogenic transformation and in what conditions these cells eventually become transformed. Nonetheless, these cells fulfill several criteria that accurately define how CSCs behave. In the sections below, the lungs and their resident epithelial cells, and how carcinogenesis can be triggered in those cells are briefly described, followed by a comprehensive description of how each resident cell proliferates and differentiates based upon the signaling pathways and chemical gradients they use during homeostasis maintenance and injury repair. This is important to recognize tissue- and cell-specific behavior that can be directly reflected towards the understandings of specific lung carcinomas.

### 2.1. Lung Tissue, Progenitor Cell, Cancer Stem Cell, and Injury-Triggered Carcinogenesis

**Figure 1:**
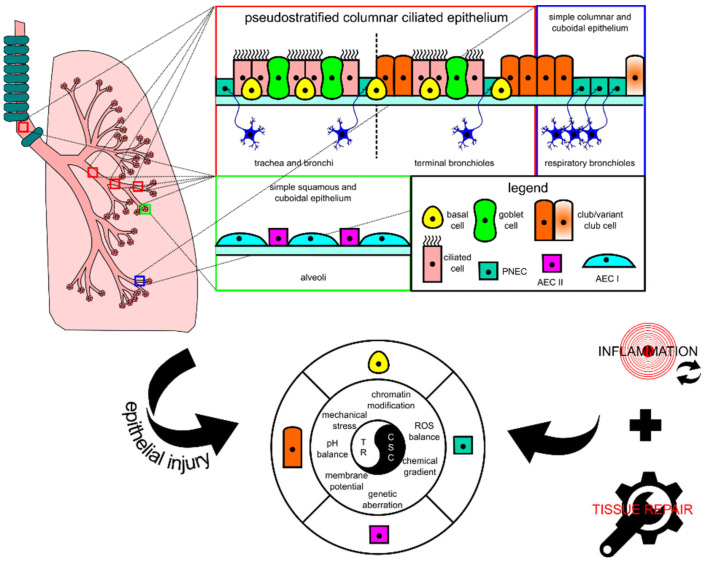
Schematic diagram showing the histological and cytological variations in different locations of the lung. In each tissue, there is at least one type of progenitor cell that is capable of proliferation, facilitating tissue repair in case of injury. In lung injury-triggered carcinogenesis, repeatedly induced inflammation accompanied by tissue repair could potentially lead to a chronic state that changes various extracellular and intracellular modulators and consequently encourages the emergence of lung cancer stem cells (CSCs) from progenitor cells. CSCs and tissue resolution (TR) are two sides of the same coin. AEC, alveolar epithelial cell; PNEC, pulmonary neuroendocrine cell; ROS, reactive oxygen species.

The lung tissue consists of respiratory epithelia that line the trachea, bronchi, bronchioles, and alveoli for gas conduction and exchange (see Figure 1 for schematic representation). Major resident epithelial cells, such as goblet cells, ciliated cells, basal cells, and innervated PNECs, are found in the pseudostratified columnar ciliated layer of the epithelia, whereas club cells are found in the bronchiolar region [9,14]. Variant club cells that are resistant to the cytotoxic effects of naphthalene reside in the respiratory bronchioles along with PNECs clustered as the neuroepithelial bodies (NEB) and in the bronchioalveolar duct junctions [9,14]. AEC I and AEC II cells are restricted to the alveolar space [9,15]. Because the lungs are constantly exposed to pathogens and irritants via respiration, the resident epithelial cells have to ensure clearance of contaminants for homeostasis maintenance and inflammation evasion.

When the lungs suffer an injury, several lung resident cells such as basal cells, club cells, variant club cells, and AEC II cells could act as facultative stem cells to repair damage by repopulating lost cells [9]. PNECs can also take part in repopulating ciliated and club cells in naphthalene-induced injury [16]. These cells’ activity is lineage-committed, which means that their renewal function is induced at the respective location where they are residing. However, recent progress in the identification of progenitor cell candidates for lung injury repair points to the involvement of other cells, such as the lineage-negative epithelial progenitor (LNEP) cell, a basal cell progenitor termed the bronchial epithelial stem cell (BESC), and a subset of the AEC II cell population termed alveolar epithelial progenitor (AEP), in repairing distal lung epithelium [17,18,19]. These cells together with the well-studied facultative stem cells are key candidates for the emergence of CSCs because their proliferative activity is mostly injury-triggered and their activity coincides with other tumor-supporting events that occur after injury consisting of DNA damage and tissue repair-associated inflammation. Given the unique cellular turnover rate of lung epithelium and potent repair capacity during injury, these cells fulfill several premises to be defined as lung CSCs: (1) they are long-lived; (2) they are mostly quiescent; (3) they can survive mild or severe injury; (4) they are known to have the potency to differentiate and proliferate for both homeostasis and injury repair [9]. Based on the similarities in the location and protein markers, lung progenitors as key players for tissue repair have clear connections with CSCs of lung carcinomas, thus explaining the overlapping between tissue repair and carcinogenesis.

Continuous lung injury disrupts the normal physiology of the lungs by impairing the morphostatic field through systemic response such as chronic inflammation, which also damages DNA and proteins. Morphostatic components may include epigenetic sets of pre-programmed modules that determine cell fate decisions and tissue organization, including chromatin modifications, bioelectric events, mechanical stress, and chemical gradients [20]. Chronic inflammatory microenvironment may eventually result in wounds that do not re-pattern properly during wound repair, which is assumed to non-autonomously encourage CSC development and support tumor initiation [21]. Intracellular damage such as in DNA can be induced by genotoxic agents or through excess reactive oxygen species (ROS) generation also produced by inflammatory cells during injury-associated lung inflammation [22]. Prolonged DNA damage may lead the progenitor cells to accumulate genetic aberrations over time which could lead to tumorigenesis, and together with the epigenetic components, they may shape how DNA aberrations are recurring in a tissue-specific manner to maintain stemness, which also non-autonomously enhances tumor progression [21]. Thus, the emergence of CSCs is achieved through a series of processes that arrest tissue repair resolution and disrupt the morphostatic field, which enables the continuous proliferation of unregulated progenitor cells.

### 2.2. Normal Physiological Responses for Homeostasis and Injury Repair in Lung Tissue

To further understand how CSCs hijack the normal stem cell machinery for proliferation and differentiation, below we comprehensively describe the processes of homeostasis and how injury repair activities differ across different progenitor cells within the lung epithelia. The focus of the description is on morphostatic signaling pathways that regulate repair processes to maintain homeostasis, and their potential to control CSC activity.

At steady state, the lung tissue is actively maintained by the Hedgehog (Hh) pathway to remain in a quiescent state. During acute lung tissue injury, Sonic Hedgehog (SHH) protein is downregulated in SHH-expressing cells including club, ciliated, and AEC II cells, which consequently induces proliferation of adjacent GLI1^+^ SHH-responsive mesenchymal cells through a self-sufficient Platelet-Derived Growth Factor, B polypeptide (PDGFB)-dependent mechanism [23]. The absence of SHH paracrine signaling from lung epithelia removes the restriction to feed proliferative signals, i.e., FGF10 for lung epithelium regeneration [24].

#### 2.2.1. Basal Cell

The basal cell is the most essential facultative stem cell marked by sex-determining region Y-box transcription factor 2 (SOX2), tumor protein 63 (TP63), and keratin 5 (KRT5) expressions. It is able to differentiate into ciliated, club, AEC I, and AEC II cells for homeostasis maintenance and injury repair [9]. A study in mice that combined long-term lineage tracing, biophysical modeling, and single-cell molecular analyses revealed that there were two pools of basal cells, comprising stem cell and committed progenitor pools [25]. SOX2 is required for basal cell specification from naïve epithelial cells as evident by transcriptional targeting of SOX2 on TP63 basal cell marker expression [26]. Besides TP63, the transcriptional coactivator YAP1 functions to maintain basal cell stemness by directly interacting with TP63 and regulating common target genes [27]. SOX2 is also used to maintain the basal cell’s identity and promote its self-renewal through Fibroblast Growth Factor Receptor 2 (FGFR2) signaling involving the Phosphoinositide 3-Kinase (PI3K)/AKT pathway [18,28].

During homeostasis maintenance, the basal cell is stochastically fated to differentiate into luminal lineages, and basal cell loss is compensated through duplication of neighboring basal cells [29]. The basal cell accomplishes club cell differentiation by generating the NOTCH3^+^ parabasal cell, which activates NOTCH1 and NOTCH2 by jagged 1 (JAG1) and JAG2 ligands [30]. Activation of Janus Kinase/Signal Transducer and Activator Transcription 3 (JAK/STAT3) signaling by interleukin 6 (IL6) from surrounding mesenchyme inhibits Notch signaling and directs the basal cell towards ciliated cell differentiation [31]. BMP ligand secretions from surrounding mesenchyme also reduce basal cell proliferation as shown by higher BMP antagonist Follistatin (FST) expression during regeneration [32].

#### 2.2.2. Club Cell

The club cell is a prominent facultative stem cell within the bronchioles [33]. This cell is marked with SCGB1A1 marker expression and generated by basal cells and variant club cells during homeostasis and injury repair [34,35]. Similar to a basal cell, the club cell bears a proximal identity of SOX2 expression along with ciliated and goblet cells [36]. A club cell can also be regenerated by a variant club cell (SCGB1A1^+^cytochrome P450 2F2 (CYP2F2)^+^) through WNT7B-triggered paracrine stimulation of mesenchymal FGF10 resulting in simultaneous activation of Wnt/β-catenin and Notch pathways and transient Epithelial-to-Mesenchymal Transition (EMT) reprogramming in the epithelia, which in turn initiates repair [37].

For homeostasis and repair, the club cells are able to differentiate into ciliated cells by acting as transit-amplifying progenitor cells in the trachea and long-term progenitor cells in the bronchioles [38]. Alternate NOTCH2-JAG1-mediated lateral inhibition in neighboring cells directs to ciliated cell differentiation [39]. A recent lineage tracing assay and in vitro differentiation assay of purified club cells demonstrated wider club cells’ functional capability in repairing injured lung epithelia outside of the bronchiolar region by dedifferentiation into basal cells and transdifferentiation into AEC II cells to repair respective regional damage [18,40]. Interestingly, tumor protein 53 (TP53) also affects club cell proliferation and differentiation by keeping epithelial quiescence at steady state [41]. Loss of expression of TP53 leads to poorer differentiation of club cell to ciliated cell but higher dedifferentiation potential, e.g., self-renewing or dedifferentiating to a more naïve cell fate such as TP63^+^KRT5^-^ progenitor cells, which can normally be inhibited through contact inhibition imposed by a neighboring basal cell [41,42]. YAP1 overexpression phenocopies TP53 loss in a club cell [27]. This may suggest that Hippo/YAP1 signaling and TP53 are somehow interacting to achieve balance to maintain lung epithelial quiescence by controlling the tendency to proliferate and differentiate [43].

#### 2.2.3. PNEC

The PNEC is marked by Achaete-Scute homolog 1 (ASCL1), calcitonin gene-related peptide 1 (CALCA), synaptophysin (SYP), chromogranin A (CHGA), and ubiquitin carboxy-terminal hydrolase isozyme L1 (UCHL1) [16]. While it is not previously known to be derived from any progenitor cell in the lung, a PNEC can be generated by a SHH-irresponsive Twist-related protein 2 (TWIST2)^+^ mesenchymal cell on the occasion when the lung epithelia suffer naphthalene or lipopolysaccharide-induced injury [44]. Apart from renewing themselves, PNECs can repopulate club and ciliated cells in dire situations [16]. UCHL1^+^ PNECs can serve to repopulate club cells when wiped out by naphthalene treatment through a NOTCH1-dependent Notch signaling induction [45]. Transdifferentiation of PNEC to the ciliated cell uses the histone-lysine N-methyltransferase EZH2-IL6-STAT3 pathway [46]. A recent study discovered that a small number (2–4 cells) of differentiated PNECs in a cluster of NEB (a group of 20–30 PNECs) possess stem cell function, thus representing dedicated stem cells that are capable of self-renewal and differentiation under the strict guidance of genes such as transformation-related protein 53 (*Trp53*) and retinoblastoma (*Rb*) (quiescent signals) to ensure injury-dependent proliferation and control of subsequent sensitivity to dispersal and transdifferentiation signals [47].

#### 2.2.4. AEC II

AEC II is a prominent facultative stem cell in the peripheral lung epithelia. This cell is marked with the expression of pulmonary Surfactant-associated protein C (SFTPC) and mainly only self-renews and differentiates into Homeodomain-only protein (HOPX)^+^Podoplanin (PDPN)^+^Aquaporin 5 (AQP5)^+^ AEC I for homeostasis maintenance [15]. AEC II is known to have restricted regenerative capacity that only generates residents within the alveolar compartment for homeostasis and mild injury repair. To maintain the integrity of the alveolar compartment, neo-basal/basal-like cells could be generated by several precursors or intermediate progenitor cells to repair severe injury, including LNEPs, BESCs, SOX2^+^ progenitor cells, and intrapulmonary bronchial progenitor cells [17,18,48,49]. In contrast to the basal cell in the proximal region, neo-basal/basal-like cells generated by LNEPs are maintained by hypoxia-inducible factor 1-alpha (HIF1A)-mediated Notch activity and differentiate into AEC II upon Wnt/β-catenin-driven Notch blockade [17,50]. On the other hand, BESCs are also capable of differentiating to neo-basal/basal-like cells that further differentiate into AEC II or club cells in response to bleomycin-induced severe lung injury [18].

FGF10-FGFR2B signaling strength controls whether the BESC-generated neo-basal/basal-like cells are expanded or transdifferentiated to AEC II; a sufficiently high level of FGF10 biases towards AEC II differentiation, whereas subsequent FGF10 supply termination leads to AEC I differentiation [18]. The sufficiency of FGF10 feed is controlled by epithelial WNT7B feedback to airway smooth muscle cells (ASMCs), which is negatively controlled by the Integrin-Linked Kinase (ILK)-Hippo pathway for quiescence maintenance [51]. Apart from being responsive to FGF10 signaling, AEC II also proliferates in response to EGFR and GTPase KRAS signaling [52]. A single Platelet-Derived Growth Factor Receptor Alpha (PDGFRA)^+^ fibroblast can serve as the stem cell niche for an AEC II by supplying Wnt ligands that allow the cell to proliferate extensively with appropriate signals and maintain its stem cell identity, which would otherwise be differentiated to AEC I upon exiting the niche [53]. This Wnt active population (AXIN2^+^Transmembrane 4 L6 family member 1 (TM4SF1)^+^) is a distinct smaller AEC II cell population termed AEPs [19]. Under acute injury response, AEPs become Wnt autocrine-proficient, thus enabling them to regenerate lost AEC I and AEC II [53]. Like how BMP signaling influences the basal cell, the stable expression of phosphorylated mothers against decapentaplegic homolog 1/5/8 (pSMAD 1/5/8) of BMP signaling in AEC I and AEC II maintains cellular quiescence, whereas expression of antagonist FST promotes AEC II proliferation [54].

**Figure 2:**
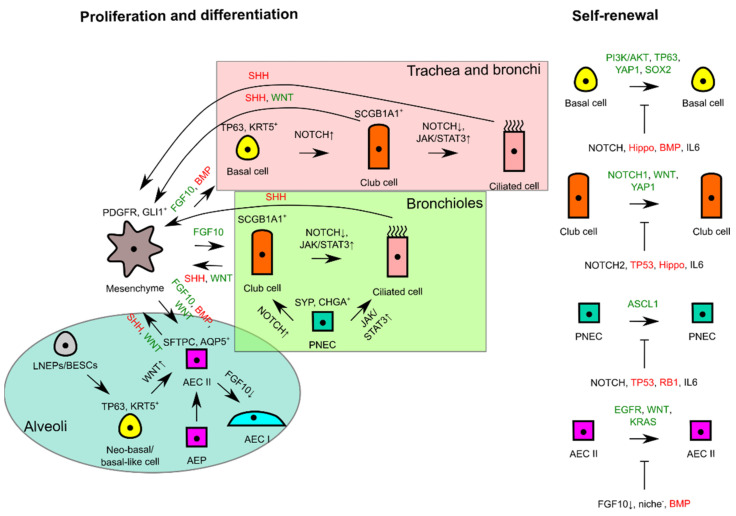
Schematic diagram illustrating self-renewal and the interactions of lung epithelial residents with mesenchyme to trigger proliferation or to maintain tissue quiescence. In the different proximodistal locations of lung epithelia, signaling pathways are used differently for differentiation, whereas similar signals are used for proliferation and lung tissue quiescence in progenitor cells of respective lung tissue locations. FGF10 or WNT from mesenchyme consequently triggers self-renewal of progenitor cells if differentiating and tissue quiescence signals (--|sign) are missing during tissue repair or homeostasis. Green signals favor proliferation, whereas red signals favor tissue quiescence. AEC, alveolar epithelial cell; AEP, alveolar epithelial progenitor; BESC, bronchial epithelial stem cell; LNEP, lineage-negative epithelial progenitor; PNEC, pulmonary neuroendocrine cell.

The signaling pathways regulating self-renewal and overall interactions of lung epithelial residents for proliferation and differentiation are simplistically summarized in Figure 2.

## 3. Dysregulations and Aberrations Supporting Lung Carcinogenesis

**Figure 3:**
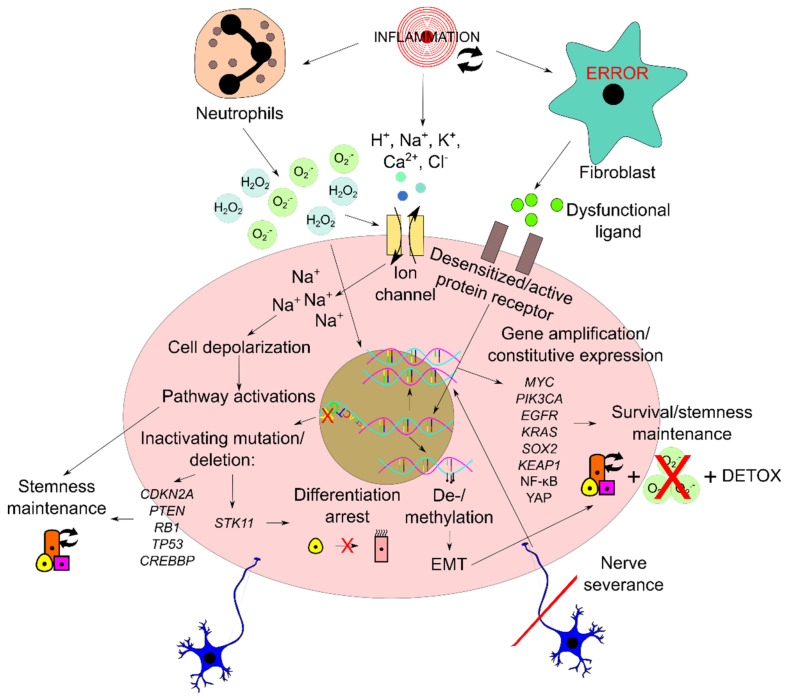
Schematic diagram depicting various molecular aberrations and dysregulations supporting the development of CSCs. The microenvironmental components are influenced by the recurrently induced inflammation and, in turn, feed the negatively affected morphostatic instructions to epithelial cells leading to aberrant changes of genetic and epigenetic components. This consequently enables favorable behavioral changes that facilitate the cellular transformation of CSCs. EMT, epithelial-to-mesenchymal transition.

Chromosomal aberrations and genetic mutations have been very well-explored as common features of malignancies, including the lung cancers. Contributions of the microenvironment have also been fairly well-documented along with the key players consisting of immune cells, mesenchymal stem cells, and fibroblasts [9]. These intrinsic and extrinsic modulators, bridged by chronic inflammation, are involved in the intricate mechanisms that underlie the lung carcinogenesis that comprises the emergence and perpetuation of CSCs. Representative molecular aberrations that cover different aspects of carcinogenesis, including survival, stemness maintenance, and evasion of quiescence/differentiation, are briefly described below. The potential impact of chronic inflammation on extracellular modulators that shape the morphostatic instructions is also concisely delineated. Additionally, the involvement of EMT in supporting carcinogenesis is also discussed. Figure 3 summarizes the variety of aberrations and dysregulations that support the transformation of progenitor cells into CSCs.

### 3.1. Molecular Aberrations Driving Tumor Initiation and Lung CSC Maintenance

The increased understanding of the molecular processes involved in programming normal lung tissue into desired lung carcinoma, irrespective of cell origin, has provided the knowledge required for dissecting molecular aberrations for the development of lung carcinomas. A mouse model with phosphatase and tensin homolog gene (*Pten*) and cyclin-dependent kinase inhibitor 2a and 2b genes (*Cdkn2ab*) deletions in addition to SOX2 overexpression in tracheobronchial basal cells, and other peripherally located club cells and AEC II cells, has been shown to induce lung SCC [55]. On the other hand, common sets of oncogenic drivers consisting of dominant negative TP53, myristoylated AKT serine/threonine kinase 1 (myrAKT1), retinoblastoma-associated protein-short hairpin RNA (*RB1*-shRNA), MYC proto-oncogene protein (MYC), and B-cell lymphoma 2 apoptosis regulator protein (BCL2) were demonstrated to transform normal human bronchial epithelial (NHBE) cells into small cell neuroendocrine cancer that had a similar transcriptional and chromatin accessibility landscape to SCLC [56]. For ADC, Notch signaling activation was shown to generate a tumor in addition to GTPase KRas activation in AEC II cells; forced activation of Notch signaling, likely NOTCH2, to block SOX2 activity was shown to transform club cells that were not previously known to progress to ADC [39,57]. Usually, these molecular aberrations act to maintain potency to proliferate, arrest differentiation, prevent quiescent restoration, and increase survival in the CSC compartment. In agreement with how the normal lung tissue operates, several tumor suppressor genes that are involved in quiescence maintenance such as *CDKN2A* that codes for p16 protein, *RB1*, *PTEN*, and *TP53* are commonly lost in lung carcinomas [58]. Also consistent with the regulators of proliferation we described above, some common gene amplification or overexpression targets found in lung carcinomas are *SOX2*, *MYC*, phosphatidylinositol-4,5-bisphosphate 3-kinase catalytic subunit alpha (*PIK3CA*) that codes for a subunit of PI3K, *EGFR*, and *KRAS* [58]. Many more molecular aberrations have recently been discovered to help us understand further how tumorigenesis in the lung can be evoked and maintained.

An example of dysregulation of an important cell oxidant-protective mechanism was demonstrated recently to promote lung tumorigenesis. Mutations in Kelch-like ECH associated protein 1 (*KEAP1*) were shown to activate the KEAP1/Nuclear Factor erythroid -related factor 2 (NRF2) pathway and subsequently promote cellular detoxifying and antioxidant activities which contributed to tumor development [59].

Nuclear Factor kappa-light-chain-enhancer of activated B cells (NF-κB) is also one of the overexpressed protein targets in NSCLCs. In a study employing CSC-enriched cisplatin-resistant NSCLCs, NF-κB expression could be reduced using a small molecule inhibitor dehydromethylepoxyquinomicin (DHMEQ) to restore sensitivity towards cisplatin treatment [60]. This indicates that the NF-κB pathway may be involved in the stemness maintenance of NSCLCs.

YAP that is constitutively inhibited by the Hippo pathway in lung progenitor cells was lately found to be overexpressed in lung ADC [61]. YAP overexpression was associated with proliferation and autophagy in lung ADC cell lines A549 and H1299 and induced activation of the AKT/mammalian Target of Rapamycin (mTOR) pathway by PTEN suppression. Thus, lung CSCs can breach the Hippo pathway’s cell-intrinsic quiescence regulating function by overexpressing YAP protein.

CREB-binding protein (*CREBBP*) is a gene encoding an acetyltransferase that is often lost in SCLC [58,62]. Such loss accelerated neuroendocrine tumors in autochthonous mouse models, including SCLC. Mechanistically, *Crebbp* was found to positively regulate cadherin 1 (CDH1) expression by acetylating histone residues, which suppressed tumorigenesis through inhibition of proliferation. Hence, CREBBP is an important negative regulator of proliferation to limit the emergence of CSC in the lung tissue.

Serine/threonine kinase 11 (*STK11*) is also one of the most frequently mutated genes in lung ADC [58]. Interestingly, mutations in this particular gene often co-occur with mutations of *KRAS* in NSCLCs [63]. Whether *STK11* was presented as single or double mutations with *KRAS*, it was associated with worse overall survival (OS) and progression-free survival (PFS) in metastatic NSCLCs. In a separate study, STK11 was identified as the normal program of ciliated differentiation in the airways which functions by suppressing Extracellular signal-regulated Kinase 1/2 (ERK 1/2) activity in mediating inactivation of pRB [64]. The loss of the differentiation program by mutations in NSCLCs explains how differentiation can be arrested in CSCs.

### 3.2. Dysregulations of Signals from Microenvironment during Lung Tissue Repair

Persistent airway inflammation observed in smoking patients with chronic obstructive pulmonary disease (COPD) and lung cancer is related to continuous tissue repair and perhaps coupled with continuous damage induction, which can be from the original inducer, e.g., cigarette smoking, or from chronic inflammation [65,66]. Chronic inflammation is a detrimental event because it can cause excessive tissue damage and disrupt the process of tissue repair. For example, cigarette smoke’s ROS might act as a second messenger signal for intracellular signaling such as the NF-κB pathway that induces inflammation or indirectly by imposing tissue damage that would also trigger inflammation as a tissue repair mechanism [67]. Cigarette smoking guarantees repeated exposure of the lung to toxic particulates and ROS, which ensures sustained inflammation. Indeed, a study found that cigarette smoke could delay the spontaneous death of neutrophils, which prolongs the tissue destruction and pathogen-killing phase through more enzymes and ROS release [68]. On the other hand, cigarette smoke can also exhibit an immunosuppressive effect on the activity of inflammatory cells responding to tissue damage [67]. Although they are increased in number, alveolar macrophages with cigarette smoke exposure have been documented to have impaired function particularly in resolving repair, thus prolonging inflammation [69]. Moreover, cigarette smoke can hinder tissue repair activity by impairing the proliferation of fibroblasts, hence compromising their tissue regenerative support function [70]. Regardless of the effect from cigarette smoke, progenitor cells need to communicate to the inflammatory cells that they are ready to expand themselves or otherwise inflammation cannot be resolved. This necessity is evident in mice with the absence of Yap/tafazzin (Taz) in the Hippo pathway; the mice displayed a delay of regenerative proliferation in AEC II and inability to upregulate NF-κB inhibitor alpha (NFKB1A) to inhibit NF-κB-mediated inflammatory response causing prolonged infection-induced inflammation [71].

Chemical signals originating from the extracellular milieu may also be negatively perturbed by chronic inflammation. Upon recurrent cycles of acute inflammation, proliferative progenitor cells of the lung in the resolution phase may be irresponsive to external quiescence and/or differentiation signals such as BMP ligands (quiescence signal), Notch ligands, and IL6 (differentiation signal) due to desensitization of receptors that transduce the signals [47]. On the other hand, it is plausible that the source of external signals, i.e., stromal cells, may be damaged in the same way as parenchymal cells by lung cancer risk factors or chronic inflammation itself, hence producing dysfunctional ligands that are incapable of activating differentiation or restoring quiescence. The BMP pathway noted as a quiescence maintaining-pathway is likely dysregulated in lung carcinomas in this way. Indeed, when the recombinant proteins of BMP6 and BMP7 ligands were treated in NSCLC and SCLC cell lines, respectively, proliferation was inhibited in those cells [72,73]. Alternatively, repeated or continuous chemokine and cytokine secretions at the inflammatory site could dilute other signals derived from the microenvironment. Chronic inflammation may also, through acute cytokine secretion such as IL1B, maintain stemness by inducing chromatin modification in *CDH1* and subsequent EMT reprogramming [74].

Aside from ligand-induced signaling through, for example, WNT7B and FGF10, cells can communicate via bioelectric signals that involve the cellular influx and efflux of H^+^, Na^+^, K^+^, Ca^2+^, and Cl^-^ ions regulated by ion channels and cause changes in membrane potential [75]. Cells in a specific region can present a spatiotemporal distribution of voltage gradients that confer a physiological state or phenotype. Bioelectric events involving changes in ion flows, pH, membrane potentials, and electric fields in the extracellular site affect morphogenetic and morphostatic instructions of tissue and regulate proliferation, differentiation/dedifferentiation, apoptosis, and migration/orientation [75,76]. In general, depolarization of cells from the initial steady state of quiescent cells causes activation of signaling pathways that can modulate various cellular processes [76]. Bioelectric signals also may be used for cell–cell communication tools through intercellular gap junctions and provide positional information and cell identity in tissue regeneration [77].

How chronic inflammation can lead to perturbation of this signaling remains unclear. However, voltage gradients that regulate tissue organization may be modified during chronic inflammation to result in hyperproliferation, which enables tumorigenic transformation. For instance, ROS from inflammatory cells such as second messenger hydrogen peroxide (H_2_O_2_) also regulates transepithelial potential and electric field to stimulate regeneration. In ROS-depleted condition by inhibition of Nicotinamide Adenine Dinucleotide Phosphate (NADPH) oxidase-mediated ROS production, regeneration was reduced in a tail-amputated *Xenopus laevis* tadpole but could be rescued by exogenously added H_2_O_2_ that was proposed to act upstream of voltage-gated Na^+^ channels to modulate regeneration [78]. This shows that chronic inflammation might amplify the regenerative activity of progenitor cells due to multiplied ROS production. Such ROS-dependent induction of regeneration may be related to an evolutionary-conserved phenomenon termed apoptosis-induced proliferation (AiP), which is a compensatory mechanism of undead or dying cells to stimulate proliferation either through ROS-tumor necrosis factor (TNF)-c-Jun N-terminal kinase (JNK)-dependent pathway or secretion of mitogenic signals such as WNT and SHH from dying cells, respectively [79].

In another instance, when apoptotic neutrophils are not cleared off by efferocytosis during chronic inflammation, their cellular contents including cytotoxic proteases, caspases, and ROS could leak to the inflammatory site [69]. Leaked ROS could be a charged molecule such as superoxide anion (O_2_^−^), which could result in unintended changes in epithelial transmembrane potential or even trans-epithelial potential [80]. Changes in transmembrane potential at the individual cell level can encourage tumorigenesis, and this is exemplified by cell depolarization in *Xenopus laevis*’s embryonic stem cells that can stimulate melanocytes to adopt a hyperproliferative invasive phenotype, which suggests a neoplastic-like transformation [81]. Indeed, one study documented that persistent inflammation could increase Gamma-Aminobutyric Acid (GABA)-induced depolarization of rat cutaneous dorsal root ganglion neurons in vitro [82]. On the contrary, hyperpolarization renders the cells resistant to malignant transformation even with oncogenes expression [83]. Future works need to confirm whether chronic inflammation can contribute to the increased depolarization of lung epithelial cells and the mechanism behind this route of tumorigenesis.

Additionally, due to the damaging nature of chronic inflammation, surrounding supporting structures such as the nerve, which functions to communicate long-range bioelectric positional and identity cues, could be cut off, which leads to interruption of the communication. Indeed, as reported in the insect *Leucophaea maderae*, severance of recurrent nerves led to a high incidence of tumor development [84]. In the lung, this situation can be especially relevant for PNECs as they are known to be innervated.

Chronic inflammation is undoubtedly involved in some dysregulations in the repair of lung epithelia during injury. Dysregulations of extracellular signaling cues partially or completely disconnect the communication effort among the regenerative players within the microenvironment, thus the tissue never gets properly repaired and leads to wounds that do not heal.

### 3.3. Cellular Reprogramming Involving Transitioning to Mesenchymal Phenotype

EMT is a normal cellular reprogramming pathway that also occurs during tissue repair, especially at the proliferative phase to encourage regenerations [37]. Our previous review has discussed the evidence of stemness induction in lung CSCs through the EMT process [9]. EMT switches on the mesenchymal phenotype with the expression of cadherin 2 (CDH2/N-cadherin) and switches off the epithelial phenotype by downregulating CDH1 as the hallmarks of activation. Many signaling pathways have been characterized to induce EMT by activating one of the three EMT-associated transcription factors consisting of zinc finger protein SNAIL, TWIST, and Zinc finger E-box-binding homeobox (ZEB); however, only Transforming Growth Factor Beta 1 (TGFB1) induction through the TGFβ pathway has the versatility to activate all three transcription factors [9]. Due to this versatility, TGFB1 can be used to abuse EMT reprogramming by CSCs because TGFB1 is one of the cytokines that are freely available during the regenerative phase [85]. Although EMT is usually transiently activated during tissue repair by chromatin remodeling that silences *CDH1*, partial EMT induction is a common sight in many cases of carcinomas, which means that it could be very well-sustained [86]. Moreover, EMT “memory” can be created by acute exposure of proinflammatory cytokine IL1B to induce sustained EMT and stemness maintenance which supports CSCs’ activity in disease progression, hence leading to common clinical outcomes such as therapeutic resistance, metastasis, and even immune evasion [74,86].

## 4. Current Use of Phytochemical Compounds in Treating Lung Cancer

Phytochemical compounds have been used in cancer treatment since the inception of chemotherapy. Vinca alkaloids were some of the earliest developed phytochemical-based compounds, including vinorelbine that was isolated from rosy periwinkle, first produced in 1979 and approved by the Food and Drug Administration (FDA) in 1994 to treat NSCLC patients [87,88,89]. In the 1960s, a new diterpenoid phytochemical called paclitaxel was discovered and isolated from the bark of Pacific yew and was approved for the use of NSCLC patients in 1998 [90]. Soon after due to compound scarcity and difficulties in isolation, a semi-synthetic drug called docetaxel was made and isolated from the needle of European yew, and it was approved in 1999 to manage NSCLC patients [87,91]. Etoposide, which was approved along with cisplatin by the FDA as a first-line therapy for SCLC in 1985, was discovered as a semi-synthetic compound of podophyllotoxin from American mandrake [92,93].

Both vinorelbine and the taxanes (paclitaxel and docetaxel) work by affecting the dynamics of microtubule polymerization. Apart from having different binding sites, they are in huge contrast to each other in affecting the polymerization of microtubules. Vinorelbine binds to tubulin dimers (α-and β-tubulin) and inhibits their polymerization into microtubules required for mitotic spindle formation [88,89]. The taxanes bind to β-tubulin and enhance the polymerization into stable microtubules thereby preventing microtubule depolymerization [90,91]. The interrupted dynamics of microtubules polymerization by those compounds leads to mitotic arrest at metaphase which results in cell death. Etoposide is known as a topoisomerase poison and it works by binding to topoisomerase II enzyme–DNA complex preventing religation of double-strand breaks (DSBs) and effectively trapping the intermediate reaction at enzyme–DNA complex during the unwinding function of topoisomerase II causing inhibition in replication fork progression and lethal DSBs [94].

These phytochemical-derived chemotherapy drugs have two major limitations. Firstly, these drugs have unwanted side effects of not only impairing DNA replication and transcription in cancer cells but also proliferative normal cells, such as hair follicle cells, gastrointestinal cells, and hematocytes leading to hair loss, weight loss, fatigue, neutropenia, leukocytopenia, thrombocytopenia, nausea, and vomiting [88,89,91]. Secondly, drug resistance is observed for these drugs mediated by expression of efflux pumps such as ATP Binding Cassette (ABC) transporter protein, defects of apoptotic pathways, mutations of drug targets that alter drug–target binding affinity, and higher DNA repair efficiency in vinorelbine, etoposide, and paclitaxel-resistant lung cancer cells [95]. These two main problems may be recurring problems for new drug development, so the underlying factors causing these should be taken into account.

## 5. The Potential Use of Phytochemical Compounds for Prevention and Targeting CSCs in Lung Cancer

Phytochemical compounds still provide good sources for drug candidates due to their limitless biodiversity, although the limitations noted above can be recurring. The formulation of the drugs can be optimized, and the active component can be used as a pure compound or be produced as a semi-synthetic or synthetic drug. Coupled with better targeting strategies, expected limitations can be overcome. To illustrate the cell-protective and anticancer activities of phytochemicals, nine phytochemical compounds will be discussed with the most active research in the field in the last decade, namely curcumin, resveratrol, quercetin, epigallocatechin-3-gallate, luteolin, sulforaphane, berberine, genistein, and capsaicin. The phytochemical compounds’ effects on signaling pathways are summarized in Table 1.

### 5.1. Curcumin

Curcumin is a polyphenolic compound isolated from the rhizome of the plant *Curcuma longa* (also known as turmeric) that is widely used as a cooking spice in Southeast Asia, India, and China. Being one of the most actively tested phytochemical compounds in the field, curcumin has been known to be an anti-inflammatory, antioxidant, and anticancer agent [96].

Curcumin’s chemopreventive effect can be linked with its antioxidant properties in vivo by boosting the expression of cytoprotective enzyme heme oxygenase 1 (HO1) that leads to the catalysis production of antioxidants [97]. Additionally, curcumin prevents inflammation by blocking cadmium-induced NF-κB-dependent IL6 and ERK-dependent IL8 epithelial secretions [98]. On the other hand, curcumin can exert its anticancer property through the inhibition of several signaling pathways. Curcumin induces apoptosis and autophagy in NSCLC by inhibiting the PI3K/AKT/mTOR pathway that is known as a downstream target of FGF signaling [99]. Hepatocyte Growth Factor (HGF)-induced EMT can also be abolished through inhibition of this pathway [100]. Wu and co-authors reported that curcumin is capable of inhibiting lung cancer growth through NOTCH1 inhibition [101]. The JAK2/STAT3 pathway was also reported to be curcumin’s target [102]. Besides targeting the PI3K/AKT pathway, curcumin can also target another more universally used paracrine or autocrine pathway in lung tissue, i.e., the Wnt/β-catenin pathway [103]. The same study also suggested SHH pathway inhibition as curcumin’s anti-CSC mechanism.

Curcumin has been associated with some limitations, including low oral bioavailability and genotoxicity when a high dose was given [104]. Systemic treatment may not be an option considering its multiple pathway-targeting activities. Low dose curcumin even for the long-term, has been found to eliminate curcumin’s carcinogenic problem and helped to alleviate therapy resistance opening the possibility for combination with other treatments [105,106]. To solve the bioavailability issue, a technique employing inhalable curcumin composite particles for direct lung delivery was recently introduced [107].

### 5.2. Resveratrol

Resveratrol is a polyphenolic stilbene compound belonging to the phytoalexin class that is derived from grape seeds and skin, and its products such as red wines. This well-studied phytochemical compound is known to exhibit antioxidant, anti-inflammatory, and anticancer activities [108].

Resveratrol’s antioxidant activity is related to its ability to decrease the oxidative status (lipid peroxidation, myeloperoxidase (MPO), xanthine oxidase (XO) activities, and 8-hydroxydeoxyguanosine level) while it enhances enzymatic and non-enzymatic antioxidants (catalase (CAT), superoxide dismutase (SOD), glutathione peroxidase (GPX) activities, total oxidant status, and thiol level) in nicotine-treated rats [109]. Its chemopreventive anti-inflammatory activity was shown in the same report by its ability to reduce IL2, IL6, and TNF release. Suppression of inflammation through the NF-κB pathway may be related to its ability to decrease the phosphorylation of AKT [110]. Resveratrol’s anticancer activity was shown by its ability to perturb STAT3 signaling and to block TGFB1-induced EMT [111,112].

Due to its mild nature, resveratrol is often used in combination with other drugs; curcumin with its wide medicinal properties may potentially be used together with resveratrol to modulate the biophysical property of cancer cells, i.e., by inducing hyperpolarization [113]. Enhanced killing of etoposide to NSCLC cells was modulated by resveratrol through downregulation of ERK1/2 and AKT pathways that promote DNA damage repair [114]. At present, resveratrol is still presented with similar limitations with other phytochemical compounds, such as some general toxicity when used excessively in high doses, genotoxicity, and low oral bioavailability [108]. To counter those problems, resveratrol must be delivered at the right concentration, and this has been addressed in the recent development of nanoparticles or nanocomposite delivery methods [115].

### 5.3. Quercetin

Quercetin is a flavonol commonly found in food ingredients such as broccoli, onions, tea, berries, and citrus fruits. It is known as a phytochemical compound that possesses antioxidant, anti-inflammatory, and anticancer properties [116].

Quercetin’s chemopreventive anti-inflammatory effect was shown by its activity in reducing benzo[a]pyrene diol epoxide (BPDE)-induced NHBE cells transformation through inhibiting IL6-dependent STAT3 activation of the epithelium and BPDE-stimulated IL6 secretion by inhibiting the NF-κB and ERK pathways in the human lung fibroblasts [117]. Quercetin’s antioxidant activity may not have been reported in the last decade in lung cancer, but it is widely known that quercetin has a positive impact on the level of glutathione (GSH) and other enzymatic antioxidants, which potentially leads to a chemopreventive effect against oxidant-induced lung cancer [118]. The proliferation of NSCLC can be hampered by quercetin through inhibition of pAKT expression by quercetin leading to cell cycle arrest [119].

Having a similar situation with resveratrol, the mild effect of quercetin requires potentiation of other drugs or therapy to work optimally. For example, a combination strategy that employed quercetin and curcumin was able to increase acetylation of TP53 which would otherwise be depressed due to benzo[a]pyrene (BP) treatment, suggesting the potential of reactivation of suppressed quiescence or apoptotic module with the phytochemical compounds [120]. With the common shortcoming of low oral bioavailability, inhalable nanoemulsion was developed to improve the delivery of quercetin to the lung tissue [121].

### 5.4. Epigallocatechin-3-Gallate

Epigallocatechin-3-gallate (EGCG) is a polyphenolic compound belonging to the catechins grouping that is most abundantly found in green tea. EGCG is yet another phytochemical compound that is known to have antioxidant, anti-inflammatory, and anticancer functions [122].

Antioxidant activity of EGCG has been ill-reported in lung cancer in the last decade. However, EGCG is well-recognized as an antioxidant phytochemical compound due to its reducing phenol rings. EGCG’s anti-inflammatory property was demonstrated by its effect in alleviating paraquat-induced acute lung injury by inhibiting the NF-κB pathway and downstream cytokine production [123]. The preventive mechanism in smoking-associated NSCLC was evident by a study showing EGCG’s action in downregulating pro-inflammatory cyclooxygenase 2 (COX2), AKT, ERK, and HIF1A in nicotine-treated cells [124]. Evidence of EGCG’s anticancer mechanisms is based on several lines of study. Importantly, EGCG was able to modulate microRNA expressions to inhibit the activity of several signaling pathways such as MAPK, PI3K, Wnt, SHH, and TGFβ pathways to inhibit NSCLC’s growth and proliferation [125]. Downregulation of Wnt/β-catenin activation decreased CSC marker expression, suppressed proliferation, and evoked apoptosis [126]. In another study, EGCG was found to downregulate the pEGFR, pAKT, pERK1/2, and p-mTOR pathways [127]. The JNK pathway was found to be another target of EGCG to induce cell cycle arrest [128]. EGCG exerted pro-oxidative property by modulating ROS level to inhibit ERK1/2 pathway [129].

Apart from the activities of EGCG as a single targeting agent, the activity of DNA methyltransferase (DNMT) and histone deacetylase (HDAC) could be hampered by EGCG and cisplatin co-treatment resulting in increased expression of genes involved in maintaining cellular quiescence [130]. Combination with quercetin was reported to potentially increase the cellular absorption of EGCG into NSCLC cells [131]. EGCG is currently presented with the common low oral bioavailability problem; thus, a technique has been developed to improve its concentration in the lung tissue using nanoemulsion [132].

### 5.5. Luteolin

Luteolin is a polyphenolic compound commonly found in tea, celery, broccoli, and green pepper. This phytochemical compound also exhibits wide medicinal value, including anti-inflammatory, antioxidant, and anticancer activities [133].

To counteract inflammation, luteolin was found to decrease HIF1A and COX2 promoter activities; reduce IL1B, IL6, IL8, and TNF secretions; and inhibit the AKT, MAPK, NF-κB, and STAT3 pathways, which prevented the malignant transformation of human lung epithelial cells treated with hexavalent chromium (Cr(IV)) [134]. The antioxidant effect of luteolin was shown by its ability to improve the level of both enzymatic antioxidants such as SOD, CAT, GPX, and glutathione-s-transferase (GST), and non-enzymatic antioxidants such as GSH, vitamin E, and vitamin C through NF-κB pathway blockade which otherwise were decreased in BP-fed mice [135]. For its anticancer mechanism, luteolin activated the MEK-ERK and AKT pathways to exert its anti-proliferative and apoptotic activities [136]. In other situations, luteolin pre-treatment diminished TGFB1-dependent EMT induction through the inhibition of PI3K-AKT-NFKB1A-NF-κB-SNAIL pathways [137]. In an apparent ROS modulation situation, luteolin activated the JNK pathway to generate O_2_^−^ to mediate cytotoxic killing of NSCLC [138].

Like EGCG, luteolin can be used to inhibit HDAC activity to potentiate cisplatin cytotoxicity in lung cancer cells [139]. A combination of low-dose EGCG and luteolin could synergistically increase apoptosis of lung cancer cells [140]. To improve cellular uptake and targeted delivery, luteolin has been encapsulated into a model of delivery such as liposomes [141].

### 5.6. Sulforaphane

Sulforaphane is an isothiocyanate commonly obtained from cruciferous vegetables such as broccoli, cauliflower, cabbage, and kale. Like many others, this phytochemical compound also has antioxidant, anti-inflammatory, and anticancer properties [142].

As a chemopreventive agent, sulforaphane was able to inhibit tumor formation induced by tobacco smoke through disruption of IL6-dependent activation of the Notch pathway and increment of CSC properties such as deltaNP63alpha, CD133, and POU domain, class 5, transcription factor 1 (POU5F1) [143]. In its anticancer response, sulforaphane activated ERK1/2 to promote the downregulation of a pro-survival cue in NSCLC called BCL2-like protein 11 (BCL2L11), resulting in its proteasomal degradation and apoptotic induction [144]. Anoikis resistance and anchorage-independent growth were inhibited by inactivating the AKT pathway and downregulation of Catenin Beta-1 (CTNNB1) [145]. Apoptosis can be induced by stimulating the generation of ROS; however, such an effect was lower in high-EGFR-expressing lung cancer cells [146]. Thus, this indicates that sulforaphane may need to be combined with TKIs or other phytochemical compounds targeting RTK such as EGFR. In another instance whereby NHBE cells were transformed by cadmium, sulforaphane was able to exert its anti-inflammatory effect by reducing ROS level which would otherwise trigger TNF release, NF-κB pathway activation, and downstream expression of COX2 [147]. Sulforaphane’s anticancer mechanism was also found to be mediated by inhibition of DNMT and HDAC activities [148].

Sulforaphane was reported to sensitize and decrease the resistance of NSCLC towards the currently used TKIs such as gefitinib [149]. Sole administration of sulforaphane or combination with gefitinib was able to inhibit the Hh pathway by downregulating SHH, Smoothened homolog (SMO), and GLI1 [149]. Good synergism in inducing cell cycle arrest and apoptosis was also observed in the treatment of NSCLC using sulforaphane and allyl isothiocyanate, suggesting the potential of combinatory exploitation of the two isothiocyanates in lung cancer therapy [150].

### 5.7. Berberine

Berberine is an isoquinoline alkaloid compound isolated from the rhizome of the herbal plants *Coptis chinensis* (Chinese goldthread) and *Hydrastis canadensis* (goldenseal). This medicinal compound is also known to exert antioxidant, anti-inflammatory, and anticancer effects [151].

As part of berberine’s anti-inflammatory mechanisms, it suppressed multiple pathways in NSCLC including NF-κB/COX2, PI3K/AKT, vascular endothelial growth factor (VEGF)/HIF1A, and MERK/ERK [152]. Berberine’s anticancer mechanism is mediated by the downregulation of CD142 (tissue factor) in NSCLC that resulted in MAPK signaling inhibition and apoptotic induction [153]. Derivative berberine hydrochloride may also inhibit proliferation and apoptosis by abolishing the activation of JAK2 and NF-κB pathways in NSCLC [154]. Chromatin remodeling is in part involved in the berberine-dependent induction of growth arrest and apoptosis which is by suppression of HDAC activities [155]. Berberine activated the p38 MAPK pathway and dispensable ERK1/2 pathways to increase TP53 and Forkhead box protein O3 (FOXO3) expressions, which accentuated growth arrest and apoptotic inductions [156]. Berberine could inhibit TGFB1-induced EMT in NSCLC, which could potentially support CSC maintenance [157]. At a low dose, berberine induced transient cellular change that was similar to cellular quiescence [158].

In some preclinical studies, berberine combined with doxorubicin was found to have a good synergistic effect against lung cancer cells [159]. Interestingly, berberine was able to sensitize lung cancer cells to doxorubicin’s cytotoxic activity by inhibiting doxorubicin-induced STAT3 activation [159]. For improving the targeted delivery of berberine, hyaluronate/lactoferrin layer-by-layer lipid was utilized [160].

### 5.8. Genistein

Genistein is an isoflavone compound that is ubiquitously found in soy and soy-based food products such as soymilk, tofu, and tempeh. Like all other phytochemical compounds listed here, genistein is blessed with antioxidant, anti-inflammatory, and anticancer properties [161].

Genistein was reported to mediate proliferative inhibition and apoptotic induction in NSCLC through suppressive activities towards the PI3K/AKT/HIF1A/VEGF and NF-κB/COX2 signaling pathways, which enhanced the production of ROS [162]. Other reports included Hepatocyte Growth Factor Receptor (HGFR) as a target for the apoptotic induction and indicated that TP53 intracellular stability was enhanced by genistein treatment [163,164].

In some preclinical studies, genistein was assessed with other therapy forms, such as trichostatin A (HDAC inhibitor) and all-trans retinoic acid (ATRA, differentiation therapy), to synergistically enhance apoptosis in NSCLC [165,166]. To co-deliver genistein with other potential drugs, an aptamer-hybrid nanoparticle has been developed and tested in NSCLC [167].

### 5.9. Capsaicin

Capsaicin is a homovanillic acid derivative that can be obtained from chili pepper—a very popular food spice consumed by people from all over the world. Although it is not well-studied yet in recent years, some evidence indicates that capsaicin may possess favorable antioxidant, anti-inflammatory, and anticancer activities [168].

Limited evidence is available on capsaicin’s activity towards lung cancer in the last decade. In BP-treated mice, capsaicin was able to prevent the development of lung cancer by inhibiting TNF and IL6 secretions, NF-κB pathway activation, and COX2 expression [169]. Capsaicin modulated the TP53 reactivation in NSCLC to degrade HIF1A, which negatively impacted the VEGF expression, therefore discouraging tumor growth [170]. Interestingly, capsaicin was also found to activate transient receptor potential vanilloid 6 (TRPV6) of cation-channel receptor to activate the calcium-mediated calpain pathway, which consequently induced apoptosis in SCLC [171].

Capsaicin has been tested along with other TKIs such as erlotinib and gefitinib in some preclinical findings where the combinations were able to enhance cytotoxicity towards NSCLC by inactivating the AKT pathway and restoring enzymatic antioxidant activities such as SOD and CAT [172,173]. To improve targeting, nanoparticles have also been developed for capsaicin delivery [173].

## 6. Discussion and Future Perspective

Malignant diseases including lung cancer have mostly been viewed as diseases at the cellular level. In this review, we attempted to view lung cancer as a tissue-level deviation that fails to adhere to morphostatic instructions as part of the larger goal of homeostasis maintenance. Then, we described studies that may point to the potential use of phytochemicals not only for prevention but also treatment, with a particular focus on CSCs. By considering the low turnover rate of lung epithelia and how injury can trigger stem cell activity, CSCs can be transformed from the proliferative residents of the epithelia in response to repeated injury induction and chronic inflammation. Molecular aberrations are undoubtedly very prevalent in lung carcinomas, and tissue-specific programs related to proliferation, quiescence, differentiation, and survival are usually preferentially selected through the cancer evolution creating versatile CSCs. These cellular processes represent important targets for CSCs.

Conventional chemotherapy and TKIs that target proliferation are not able to effectively eliminate CSCs due to therapeutic resistance leading to tumor recurrence [9]. Differentiation therapy using retinoids has also been documented to face the same resistance problem, which limits its usage [174]. Although therapies that can restore quiescence such as CDK inhibitors have led to some disappointment in lung cancer, they are to be explored further including other alternative quiescence inducers [175,176].

With the role of ROS and inflammation in CSC emergence and maintenance being recognized, phytochemical compounds that have preventive or cell-protective activities including anti-inflammatory and antioxidant activities can potentially be utilized as prevention and therapy candidates for tackling the inflammatory and ROS imbalanced microenvironment that perpetuates CSCs, thus helping to eliminate the source of disease. Increasing evidence has demonstrated that anti-inflammatory drugs may be used as adjuvants for chemosensitization and chemoprotection against conventional therapy’s side effects [177]. It is worth mentioning that therapeutic modulating of ion channels using phytochemical compounds in lung cancer could be a future endeavor because modulating hyperpolarization to resting membrane potential can effectively restore CSCs to their quiescence [178]. Recent mounting evidence has shown that phytochemical compounds have the potential to be used as an alternative to immune checkpoint inhibitors against Programmed cell death 1 ligand 1 (PDL1) such as the cases of EGCG and berberine [179,180]. Future study is still necessary to demonstrate whether the utilization of phytochemical compounds can help alleviate the resistance challenge and side effects of immunotherapy.

While embryonic signaling pathways such as Wnt/β-catenin, Notch, and Hh, and pro-mitotic signaling pathways such as PI3K/AKT, RTKs, JNK, and JAK/STAT3 can each be a decent target for inhibiting CSCs, it is good to realize that cancers are tissue-specific diseases and therefore should be treated contextually as signaling pathways behave differently in a tissue-specific manner. For instance, the ability of curcumin and sulforaphane to downregulate the Notch pathway in lung tissue can be contradictory to the lung cancer inhibitory goal. NOTCH1 is an instructive signal for differentiation of club cells from basal cells; thus, enforced Notch deactivation may arrest differentiation and help to maintain CSCs of SCC. In the distal lung, its inhibition is likely oncogenic because Notch inhibition activates the AEC II cell differentiation program and therefore risks supporting the CSCs of ADC. Perhaps due to these reasons, Notch inhibition has achieved little success in clinical trials [9].

Moreover, inhibiting JAK/STAT3 may not be as favorable as originally thought. Half a dozen of the phytochemical compounds listed above can inhibit the JAK/STAT3 pathway. As reported in a study for curcumin, targeting the JAK/STAT3 pathway may only be suitable for ADC [102]. Like Notch, the JAK/STAT3 pathway is a less unifying pathway in lung epithelia. Inhibiting JAK/STAT3 might result in a ciliated cell differentiation blockade which enables CSCs derived from basal cells, PNECs, and club cells to maintain stemness.

Another unfavorable target in lung tissue is the Hh pathway. Although it is a unifying pathway across proximodistal regions, the Hh pathway using SHH as a ligand is a negative regulatory pathway for lung epithelial proliferation exerted from the surrounding mesenchyme; thus, once inhibited, it would result in the unfavorable therapeutic outcome of unregulated FGF10-dependent proliferation of CSCs.

Since lung tissue is mostly quiescent, targeting lung CSCs with the inhibition of locally unifying signaling pathways should not cause many issues. The Wnt/β-catenin pathway is generally used for paracrine signaling between epithelial and stromal cells and sometimes can be used in an autocrine manner. FGF2 and other RTKs that activate the downstream PI3K/AKT pathway are the response that bridges the communication between lung epithelial cells and mesenchyme. Thus, targeting the Wnt/β-catenin and PI3K/AKT pathways may yield favorable outcomes and little toxicity. NF-κB universally controls inflammation and is usually downregulated in preparation for regeneration, whereas EMT reprogramming is usually transiently activated for regeneration; therefore, they should be inhibited to restore tissue quiescence. However, one study found that inhibiting NF-κB activity may moderately promote acceleration of SCLC development, thus suggesting that targeting NF-κB may not be an option for treating SCLC patients [181]. Besides inhibiting these stemness maintaining pathways, phytochemical compounds should preferably restore the activation of signaling pathways that are active at the steady state to maintain quiescence of lung tissue, namely the Hh, BMP, and Hippo pathways. These pathways should be the preferred target of restoration since they are the inherent negative regulatory pathways of quiescence of the lung tissue. However, the stimulatory activity of the listed phytochemical compounds towards those pathways is not well-studied and hence requires further investigation.

Due to phytochemical compounds’ pleiotropic effects and complex chemical structures, their usage requires special attention. With the desire to improve them as drug candidates, several approaches for improvement can be taken. Firstly, analogs of phytochemical compounds can be synthesized by structural modifications of the original phytochemical structure to simplify the overall structure to have smaller size without unnecessary off-target functional groups, increase efficacy, increase selectivity, or improve physicochemical properties [182].

Secondly, phytochemical compounds can be loaded into drug vehicles to facilitate a more targeted transport to lung tissue. The vehicles or carriers could take several forms, including large porous microparticles, polymeric nanoparticles, micelles, liposomes, or polyethylene glycol (PEG)-conjugated agents, among others [183]. Phytochemical compounds loaded into carriers can be directly administered to the lungs by inhalation. Inhalation ensures quick response and good local bioavailability besides eliminating several challenges in drug delivery such as absorption, stability, clearance, and off-target side effects due to systemic treatment, like when using oral and intravenous administrations [184]. Besides, data obtained by in vitro testing may be more relevant to the situation during inhalation delivery.

Thirdly, efficacy and toxicity are balanced by dosing strategy. With inhalation delivery in place of the current standard drug administration, a lower dose can be administered to achieve similar efficacy without evoking toxicity in other systems. Our study revealed that the phytochemical compound chelerythrine chloride can significantly inhibit β-catenin nuclear localization at minimally cytotoxic concentrations, thus indicating that pathway activity targeting is achievable at a low dose [185]. Dosing can be adjusted to an even lower level when phytochemical compounds are co-delivered to the site of the target, e.g., EGCG and luteolin combinations could produce a synergistic effect when co-treated at a low dose [140]. Indeed, a combinatory regimen is preferred as demonstrated by how mixtures of herbal ingredients are prescribed by physicians in traditional medicine even though only a single ailment is treated [186].

Utilizing phytochemical compounds as drug candidates is associated with some practical advantages and disadvantages. Firstly, natural products including plants and their derived phytochemical compounds have been subjected to animal or self-experimentation and use for centuries, and hence there is little concern over the safety or side effects of the consumed plant products [187]. Some might even be made into a tonic for regular consumption over a long time. Secondly, phytochemical compounds are structurally diverse and unique, making them very likely to have a new chemical entity that could serve as a reference for innovative drug discovery. Thirdly, many phytochemical compounds are derived from dietary vegetables and fruits, thus providing an inexpensive supply of starting biomaterial for drug production—such as those that we have listed in this review.

However, disadvantageously, finding novel chemical entities can be laborious and time-consuming [187]. Given the time and resources needed to isolate, purify, and then examine their therapeutic value after a novel encounter, it may end up being too expensive to produce. The cost of production and availability may also depend on the rarity of the plant species or scarcity of obtainable amount from where the phytochemical compound was isolated. Fortunately, with today’s advancement of scientific knowledge, desired phytochemical compounds can be produced by bioengineering so that they are available in excess from plant sources or by bioengineering at microorganism-dependent biochemical factories to meet the economic standards of drug production [188,189].

In conclusion, plant-based diets provide added health benefits due to their extra constituent of phytochemical compounds. According to recent research, these compounds possess anticancer, antioxidant, and anti-inflammatory activities that may not only be useful in the prevention but also in the treatment of lung cancer. Besides these aspects, potential targets that should be explored further include ion channels and quiescence pathways. When targeting signaling pathways, it should either be tissue-specific or more locally unifying, depending on the target carcinoma. Although lung cancer treatment with phytochemical compound-derived chemotherapies has had some downsides, with better strategies and targets, newer phytochemical-derived drugs can be developed into successful therapies.

## Figures and Tables

**Table 1:** Summary of the activity of listed phytochemical compounds on targeting signaling pathways.

Phytochemical	Antioxidant	Anti-Inflammatory	Signaling Pathway Target								
			Wnt	Notch	Hh	JNK	JAK/STAT3	PI3K/AKT	NF-κB	RTK	TGFβ/BMP
Curcumin	Yes	Yes	↓	↓	↓	-	↓	↓	↓	↓	-
Resveratrol	Yes	Yes	-	-	-	-	↓	↓	↓	↓	↓
Quercetin	Yes	Yes	-	-	-	-	↓	↓	↓	↓	-
EGCG	Yes	Yes	↓	-	↓	↓	-	↓	↓	↓	↓
Luteolin	Yes	Yes	-	-	-	↑	↓	↑↓	↓	↑↓	↓
Sulforaphane	Yes	Yes	↓	↓	↓	-	-	↓	↓	↑↓	-
Berberine	Yes	Yes	-	-	-	-	↓	↓	↓	↑↓	↓
Genistein	Yes	Yes	-	-	-	-	-	↓	↓	↓	-
Capsaicin	Yes	Yes	-	-	-	-	-	↓	↓	-	-

## Data Availability

Not applicable.
